# Use of faecal immunochemical tests common in patients with suspected colorectal cancer but unrelated to travel distance to secondary care: a population-based study from Swedish primary care

**DOI:** 10.1080/02813432.2022.2144934

**Published:** 2022-11-15

**Authors:** Cecilia Högberg, Olof Cronberg, Hans Thulesius, Mikael Lilja, Stefan Jansson, Ulf Gunnarsson

**Affiliations:** a Department of Public Health and Clinical Medicine, Unit of Research, Education and Development Östersund, Umeå University, Umeå, Sweden; b Department of Clinical Sciences, Lund University, Lund, Malmö; c Department of R & D, Region Kronoberg, Växjö, Sweden; d Department of Medicine and Optometry, Linnaeus University, Kalmar, Sweden; e School of Medical Sciences, University Health Care Research Centre, Örebro University, Örebro, Sweden; f Department of Public Health and Caring Sciences, Uppsala University, Uppsala, Sweden; g Department of Surgical and Perioperative Sciences, Surgery, Umeå University, Umeå, Sweden

**Keywords:** Colorectal cancer, travel time, faecal immunochemical tests, occult blood, primary health care, Sweden

## Abstract

**Background:**

Evidence is increasing for the use of faecal immunochemical tests (FITs) for occult blood as diagnostic tools when colorectal cancer can be suspected. FITs have been used for this purpose in Swedish primary care since around 2005 despite absence of supporting guidelines. To our knowledge, the extent of this use has not been studied.

**Objective:**

To investigate the use of FITs as diagnostic tools, and if the use was related to patient age, sex and travel time from primary care to diagnostic facilities in secondary care.

**Design:**

Population-based retrospective study using data from electronic health records.

**Setting and subjects:**

Patients ≥18 years that provided FITs in primary care in five Swedish health care regions during 2015. Driving times from their primary care centres to secondary care were calculated.

**Main outcome measures:**

The proportion of patients that provided FITs was calculated for each region, different age intervals and grouped driving times.

**Results:**

18,913 patients provided FITs. The proportion of listed patients in the five regions that provided FITs increased with age: 0.86–1.2% for ages <65 years, 3.6–4.1% for ages 65–79 years and 3.8–6.1% for ages ≥80 years. Differences between the regions were small. There was no overall correlation between the proportion of patients that provided FITs and driving time to secondary care.

**Conclusion:**

FITs were used extensively in Swedish primary care with a higher use in older age groups. There was no tendency towards a higher use of FITs at primary care centres with longer driving times to secondary care.

**Key Points**
- Evidence is increasing for the use of faecal immunochemical tests (FITs) as diagnostic tools when colorectal cancer can be suspected. We investigated the use of FITs in Sweden.
- FITs were used extensively in primary care especially in older age groups.
- There were small differences in the use of FITs between five studied health care regions.
- There was no tendency towards a higher use of FITs at primary care centres with longer driving times to diagnostic facilities in secondary care.

## Introduction

Symptoms suggestive of colorectal cancer (CRC) are common reasons to consult primary care [1]. However, these symptoms are seldom caused by cancer [2,3]. It can be challenging for primary care physicians to decide which patients to refer for further investigation [4]. A standardised care pathway for CRC, which includes recommendations on referral, was introduced in Sweden in 2016 [5]. However, a recently published study indicates that many CRCs are not identified *via* this pathway [6]. Evidence has earlier been scarce, but is now increasing, for the use of faecal immunochemical tests (FITs) for occult blood as diagnostic tools to aid prioritising referral and further investigations for patients when CRC can be suspected [7–11].

In the UK, Spain and Australia, the use of FITs for symptomatic patients in primary care is included in guidelines since 2017–2018 [12–14]. In Sweden, the use of FITs for patients >40 years with change in bowel habits was included in the standardised care pathway for CRC in 2022. However, tests for faecal occult blood have traditionally been used for symptomatic patients in primary care and hospitals in Sweden for many years, this in spite of the lack of nation-wide and local guidelines, the earlier lack of evidence and no coordinated education on the subject. Qualitative FITs replaced the older guaiac-based tests from around 2005 and onwards. These FITs are visually interpreted immunochromatographic tests in cassettes or dipsticks with pre-set cut-off values and give a positive or negative result. The vast majority of the FITs are requested and analysed at primary care centres.

Colonoscopy or CT colonography are used to diagnose suspected CRC. Long travel distance and travel time for patients to diagnostic facilities in secondary care might influence the possibility for and willingness of patients to attend these procedures. A Scottish study has reported that rural patients diagnosed with cancer were more likely to have had blood tests ordered by their GPs [15]. Possibly, primary care physicians working at the most remote primary care centres could be more inclined to request FITs as a part of their clinical investigation before decision on referral.

An optimal use of FITs as a diagnostic tool in primary care must be based on knowledge gathered from decision-making among primary care physicians in their handling of patients with symptoms possibly emanating from CRC. To provide an analysis of the true spectra based on routine care, data sampled prior to the introduction of standardised care pathways must be used. In a Swedish retrospective population-based cohort study including five regions we found an overall FIT sensitivity of 91,4%, a positive predictive value (PPV) of 6.8% and a negative predictive value (NPV) of 99.7% for CRC in patients aged ≥40 years [16]. An analysis of the cohort in one of the regions showed that the FIT’s diagnostic performance was similar for patients with and without a history of rectal bleeding [11]. Examples of other relevant questions to be analysed are the clinical features that resulted in the use of FITs, to what extent FITs were used, differences according to travel time to more advanced diagnostic facilities, patient age and sex, as well as regional variability.

The aim of this study was to investigate to what extent FITs were used as diagnostic tools in a population-based material from five regions in Sweden before the introduction of standardised care pathways for CRC, and if the use of FITs was related to patient age, sex and travel time from primary care centres to diagnostic facilities in secondary care. To the best of our knowledge, no such analyses have yet been published.

## Material and methods

**Figure 1.**
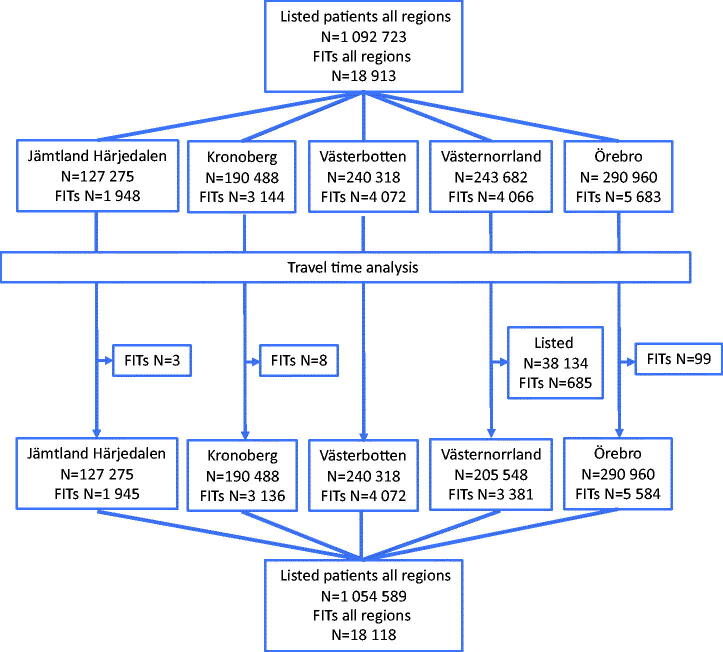
Number of listed patients and FITs provided in the different regions.

This is a post-hoc analysis of a retrospective population-based cohort study including all patients aged ≥18 years for whom FITs were requested and registered in primary care in five Swedish regions (Jämtland Härjedalen, Kronoberg, Västerbotten, Västernorrland and Örebro) during the period 1 January to 31 December 2015. Results on the FIT outcomes have been published previously [11,16]. Details on methods are published elsewhere [16]. In brief, the electronic health record system in each region (shared by that region’s primary care centres) was used to identify the patients. All primary care centres in the above-mentioned regions were included, with the exception of four centres in Västerbotten (16,048 listed patients), which had their own health record systems (Figure 1). For 685 patients, that had provided FITs in one area with five primary care centres in Västernorrland, it was not possible to safely determine at which centre they were listed. These centres and patients were omitted from the travel time analysis. For 99 patients in Örebro, 8 in Kronoberg and 3 in Jämtland Härjedalen that had provided FITs no specific primary care centre was registered, these patients were also omitted from the travel time analysis. There were no previous or on-going screening programmes for CRC in the participating regions.

As the risk of CRC increases with age, the proportion of listed patients that provides FITs can be assumed to be higher in areas with an older population. The number and age distribution of listed patients at each primary care centre for December 2015 was obtained from the regions’ health care administrations. Each region used different intervals for age registration of the listed patients; the age intervals common for all regions, and thus the intervals possible to use, were 64 years and younger, 65–79 years, and 80 years and older.

Travel time in minutes by car from each primary care centre to their respective hospital for referral was measured in ArcGIS online (Esri, Redlands, CA). We used the default setting for Rural driving time mode. The primary care centres were divided into six groups with travel times ≤15, 16–30, 31–45, 46–60, 61–120 and >120 min.

### Statistics

For statistical analysis, we used SPSS version 26 (IBM, Armonk, NY). We calculated the proportion of patients that had provided FITs for each region, each age interval and travel time group. Confidence intervals (CI) of 95% were calculated with OpenEpi version 3.01 using Wilson score [17,18].

## Results

There were 153 primary care centres in the five regions, with 786 to 21,068 (median 6222; inter quartile range [IQR] 3719–9942) patients listed at each centre, and a total of 1,092,723 listed patients (Table 1).

**Table 1.** Characteristics of the five regions included in the study.

Region	Primary care centres, * n*	Listed patients, *n*	Patients listed/PCC, * n*, median (min–max [IQR])	Patients that provided FITs, *n*	Sex of patients that provided FITs, female, %	Age of patients that provided FITs, years, median [IQR]	Driving time from each PCC to referral hospital, minutes, median (min–max [IQR])
Jämtland Härjedalen	27	127,275	3662 (786–15,249 [1523–5828])	1948	61.4	67 [51–76]	52 (3–166 [20–76])
Kronoberg	31	190,488	4576 (1642–13,228 [3322–9941])	3144	58.8	65 [49–76]	19 (1–55 [5–35])
Västerbotten	34	240,318	5084 (1562–19,082 [3504–9506])	4072	60.5	64 [47–76]	24 (2–160 [6–52])
Västernorrland	32	243,682	6958 (1268–17,257 [4486–9753])	4066	61.9	66 [50–75]	17 (2–72 [8–35])
Örebro	29	290,960	9750 (3249–21,068 [7044–12,128])	5683	59.9	64 [45–76]	10 (3–56 [7–25])
**Total**	153	1,092,723	6222 (786–21,068 [3719–9942])	18,913	60.4	65 [48–75]	21 (1–166 [8–42])

FIT: faecal immunochemical test; PCC: primary care centre; IQR: interquartile range.

In total, 18,913 (60.4% female) patients provided FITs. The median age was 65 (IQR 48–75) years. Of the patients that provided FITs, 83.6% (59.7% female) were aged 40 years and older and 72.9% (58.9% female) were aged 50 years and older. In the five regions, the percentages of listed patients that provided FITs were 0.86% (CI 0.81–0.92%) to 1.2% (CI 1.2–1.3%) for ages <65 years, 3.6% (CI 3.4–3.9%) to 4.1% (CI 3.9–4.2%) for ages 65–79 years, and 3.8% (CI 3.5–4.1%) to 6.1% (CI 5.8–6.5%) for ages ≥80 years (Table 2). The proportion of listed patients that provided FITs at each separate primary care centre during the year was 0–3.6% for ages <65 years, 0.81–14.5% for ages 65–79 years and 1.1–10.4% for ages ≥80 years.

**Table 2.** Number of patients that provided FITs in each region during 2015, stratified for age groups.

	All ages			0–64 years			65–79 years			≥80 years		
Region	Listed patients, *n*	FITS, *n*	FITs % of listed (CI)	Listed patients 0–64 years, *n* (% of all ages)	FITs, *n*	FITs % of listed (CI)	Listed patients 65–79 years n (% of all ages)	FITs, *n*	FITs % of listed (CI)	Listed patients ≥80 years, *n* (% of all ages)	FITs, *n*	FITs % of listed (CI)
Jämtland Härjedalen	127,275	1948	1.5 (1.5–1.6)	97,941 (77.0)	846	0.86 (0.81–0.92)	21,595 (17.0)	786	3.6 (3.4–3.9)	7739 (6.1)	316	4.1 (3.7–4.5)
Kronoberg	190,488	3144	1.7 (1.6–1.7)	150,002 (78.7)	1521	1.0 (1.0–1.01)	29,083 (15.3)	1085	3.7 (3.5–4.0)	11,403 (6.0)	538	4.7 (4.3–5.1)
Västerbotten	240,318	4072	1.7 (1.6–1.7)	190,978 (79.5)	2064	1.1 (1.0–1.1)	36,213 (15.1)	1350	3.7 (3.5–3.9)	13,122 (5.5)	658	5.0 (4.7–5.4)
Västernorrland	243,682	4066	1.7 (1.6–1.7)	185,961 (76.3)	1907	1.0 (1.0–1.1)	42,748 (17.5)	1590	3.7 (3.5–3.9)	14,973 (6.1)	569	3.8 (3.5–4.1)
Örebro	290,960	5683	2.0 (1.9–2.0)	229,228 (78.8)	2854	1.2 (1.2–1.3)	46,148 (15.9)	1876	4.1 (3.9–4.2)	15,584 (5.4)	953	6.1 (5.8–6.5)
Total	1,092,723	18,913	1.7 (1.7–1.8)	854,110	9192	1.1 (1.1–1.1)	175,787	6687	3.8 (3.7–3.9)	62,821	3 034	4.8 (4.7–5.0)

FIT: faecal immunochemical test; CI: 95% confidence interval.

**Figure 2.**
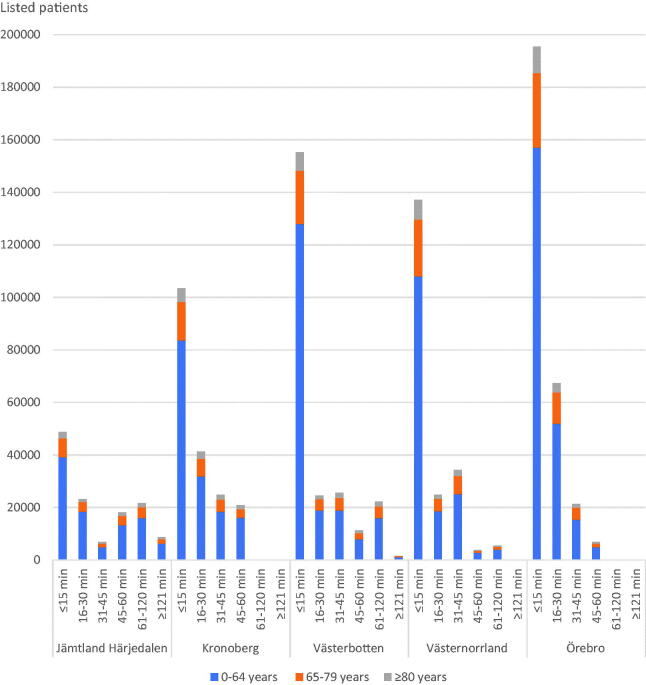
Patients’ driving times from their primary care centres to their hospital for referral, stratified for ages and the five regions.

A total of 18,118 (60.5% female) patients were included in the travel time analysis. The distances from the primary care centres to their respective hospital for referral varied from 0.4 to 232 km with driving times from one to 166 min. Driving time from their primary care centre to specialist care was 15 min or less for 61% of the patients. For 1% of the patients driving time was over 2 hours. In two of the regions, no primary care centre had a driving time over 60 min (Figure 2). The primary care centres with longer driving times had higher proportions of listed patients in the two older age groups (Table 3). The percentages of listed patients that provided FITs in the different driving time groups were 0.72% (CI 0.55–0.94%) to 1.2% (CI 1.1–1.2%) for ages <65 years, 2.9% (CI 2.3–3.7%) to 4.0% (CI 3.6–4.4%) for ages 65–79 years and 2.7% (CI 1.8–4.1%) to 5.1% (CI 4.4–5.8%) for ages ≥80 years.

**Table 3.** Number of patients that provided FITs at primary care centres, grouped for driving times to specialist care and stratified for age groups.

		All ages	<65 years			65–79 years			≥80 years		
Driving time	Primary care centres, *n*	Listed patients, *n*	Listed patients, *n* (% of all ages)	FITs, *n*	FITs % of listed (CI)	Listed patients, *n* (% of all ages)	FITs, *n*	FITs % of listed (CI)	Listed patients, *n* (% of all ages)	FITs, *n*	FITs % of listed (CI)
0–15 min	63	639,997	515,754 (80.6)	5688	1.1 (1.1–1.1)	92,054 (14.4)	3630	3.9 (3.8–4.1)	32,185 (5.0)	1598	5.0 (4.7–5.2)
16–30 min	28	181,295	139,593 (77.0)	1287	0.92 (0.87–0.97)	31,375 (17.3)	1109	3.5 (3.3–3.7)	10,327 (5.7)	499	4.8 (4.4–5.3)
31–45 min	23	112,866	82,756 (73.3)	970	1.2 (1.1–1.2)	22,003 (19.5)	796	3.6 (3.4–3.9)	8106 (7.2)	388	4.8 (4.3–5.3)
46–60 min	15	60,905	44,990 (73.9)	442	0.98 (0.90–1.08)	11,263 (18.5)	417	3.7 (3.4–4.1)	4652 (7.6)	219	4.7 (4.1–5.4)
61–120 min	15	49,245	36,096 (73.3)	376	1.0 (0.94–1.15)	9357 (19.0)	370	4.0 (3.6–4.4)	3792 (7.7)	193	5.1 (4.4–5.8)
>120 min	4	10,281	7387 (71.9)	53	0.72 (0.55–0.94)	2054 (20.0)	60	2.9 (2.3–3.7)	840 (8.2)	23	2.7 (1.8–4.1)
All driving times	148	1,054,589	826,576 (78.4)	8816	1.1 (1.0–1.1)	168,106 (15.9)	6382	3.8 (3.7–3.9)	59,902 (5.7)	2920	4.9 (4.7–5.1)

FIT: faecal immunochemical test. CI: 95% confidence interval.

## Discussion

This population-based study, including symptomatic patients in primary care in five Swedish regions, shows that FITs were frequently used as diagnostic tools for CRC especially in high ages. The use was of similar extent and with similar age and sex distribution in the regions. No tendency was found to a more generous use of FITs at primary care centres with longer driving times to specialist care.

### Strengths and limitations

This study has some strengths. It includes population-based data from five regions in different parts of Sweden with more than one million inhabitants living in cities as well as in sparsely populated areas. The coverage of the primary care centres and listed inhabitants in these regions was almost complete.

The study also has some weaknesses. As the incidence of CRC increases above ages >40–50 years, it would have been of interest to study the use of FITs in ages 40–65 years, but this was not possible because of the differences in the regions’ registration of listed patients’ ages. However, 77% of CRC cases occurred in patients aged ≥65 years in 2015 in the studied regions [19]. We were also unable to retrieve the driving times from patients’ homes to their primary care centres and to the hospitals, which would probably have provided better information on the impact of travel time. However, this study gives information on how primary care physicians use FITs in different locations.

### Findings in relation to other studies

We have found no studies on how much FITs are used in symptomatic patients in other countries for comparison. This underlines the relevance of assessing primary care physicians’ approach to patients presenting with symptoms possibly indicating CRC, and the use of FITs in the clinical handling of these patients.

In spite of a lack of national guidelines on the use of FITs, the differences between the regions in the proportion of patients that provided tests during the studied year were small. This indicates an informal national agreement based on cumulative experiences forming a common indication strategy. The variability between the individual primary care centres was larger. It seems likely that local traditions and individual physicians’ preferences could influence the use of FITs, especially in the absence of guidelines. However, some centres were small and consequently the statistical uncertainty was greater for these numbers.

Almost 5% of the listed patients aged ≥80 years provided FITs during 2015. As stated above, patients with symptoms suggestive of CRC are common in primary care and FITs were obviously a part of the primary care physicians’ tool kit of diagnostic tests. This is supported by findings in a previous qualitative study [20].

Of all patients providing FITs, 16% were under 40 years of age. In this age group, CRC is rare and in the five studied regions only 15 of 871 (1.7%) of patients diagnosed with CRC in 2015 were aged <40 years [19]. It could thus perhaps be appropriate to reduce the use of FITs in patients aged <40 years.

There was no tendency towards a higher use of FITs at primary care centres with longer driving times to diagnostic facilities in secondary care. Instead, the use of FITs was lower at the most remotely situated primary care centres. In this driving time group, 2.7% of the patients aged ≥80 years provided FITs compared to 4.7–5.1% of the patients aged ≥80 years in the groups with shorter driving times. As there were only four primary care centres in the most remote group of centres, it seems uncertain if their lower use of FITs was connected to the long distance to the respective hospital. Local traditions at these few centres may have influenced the results. A Danish study that included patients diagnosed with cancer indicated that physicians, whose patients had longer travel distances to diagnostic facilities and where cancer was not suspected, were more likely to use ‘wait-and-see’ or ‘medical treatment’ as a diagnostic strategy [21]. However, travel distances are considerably shorter in Denmark than in Sweden which makes comparisons difficult. It is also possible that patients in these very remote areas had extra-long driving times to their primary care centres and so were less inclined to seek care and provide tests. Regardless of this, a previous study from one of the most sparsely populated regions in our study found no relationship between the risk of acute surgery for colon cancer and the patient’s travel distance from home to the hospital, and another study from four sparsely populated regions in the north of Sweden concluded that there was no association between travel time and CRC survival [22,23]. This indicates an effective care of symptomatic patients also at the most remote primary care centres in Sweden. However, areas exist worldwide with longer travel distances where studies may reveal relationships to the use of FITs, and diagnosis and treatment of CRC.

### Implications for the future

It is probable that the use of FITs declined in favour of immediate colonoscopy after the introduction of the Swedish standardised care pathway for CRC, as FITs were not included in the recommended procedures until 2022. It is likely that the use will increase again as patients with change in bowel habits now are obliged to show a positive FIT result to enter the standardised care pathway. This should presumably decrease the number of colonoscopies needed for this symptom and contribute to a better use of available resources. However, patients with a positive FIT but no alarm symptom (i.e. change in bowel habit, rectal bleeding or anaemia) are not eligible for the standardised care pathway. Here is a risk of delayed or missed CRC diagnoses, as many cases of CRC present with other complaints than alarm symptoms [24]. When CRC can be suspected it seems desirable to use FITs also in patients without the mentioned alarm symptoms and for patients with a positive FIT to have easy access to further investigation.

## Conclusion

FITs to detect faecal occult blood were used extensively as diagnostic tools in Swedish primary care before the introduction of standardised care pathways for CRC with a higher use in older age groups and small differences between five studied regions. There was no tendency towards a higher use of FITs at primary care centres with longer driving times to diagnostic facilities in secondary care.

## Ethical approval

The study was approved by the Regional Ethical Review Board, Umeå University (Ethics approval 2017/451-31).
